# Optimal Puncture Number and Tissue Evaluation Method in Endoscopic Ultrasound-Guided Fine-Needle Biopsy for Patients with Malignant Neoplasm of Pancreas

**DOI:** 10.3390/diagnostics16030397

**Published:** 2026-01-27

**Authors:** Te-Jung Chuang, Pei-Tzu Chen, Jung-Chun Lin, Hsuan-Wei Chen

**Affiliations:** 1 Division of Gastroenterology, Department of Internal Medicine, Tri-Service General Hospital, National Defense Medical University, Taipei 114202, Taiwan; ronnie462006@gmail.com (T.-J.C.); leang116101@gmail.com (P.-T.C.); 2 Division of Gastroenterology, Department of Medicine, Hualien Armed Forces General Hospital, Hualien City 971051, Taiwan

**Keywords:** diagnostic rate of malignancy, macroscopic on-site evaluation, pancreatic cancer, rapid on-site cytopathologic evaluation, ultrasound-guided fine-needle biopsy

## Abstract

**Background/Objectives**: Endoscopic ultrasound-guided fine-needle biopsy (EUS-FNB) has become the standard for diagnosing solid pancreatic masses (SPMs). However, in the absence of rapid on-site cytopathologic evaluation (ROSE), the optimal number of needle passes remains uncertain. This study aimed to evaluate the diagnostic performance of EUS-FNB using a gross-eyed evaluation with the fanning technique in patients with pancreatic malignancy. **Methods**: This retrospective single-center study included 140 patients with confirmed pancreatic malignancy who underwent EUS-FNB with at least three needle passes between January 2022 and December 2025. Gross-eyed visual inspection for whitish core tissue was used to assess the specimen adequacy. The primary outcome was the diagnostic rate of malignancy. The secondary outcomes included the tissue adequacy and yield rate of malignancy. **Results**: The diagnostic rate of malignancy improved with additional passes, 72.1% for one pass, 82.9% for two, and 90.7% for three. However, the gain beyond two passes was marginal. The tissue adequacy was high across all passes (≥94.3%), with most samples deemed adequate within two passes. The yield rate of malignancy similarly improved from 82.1% (one pass) to 91.4% (three passes). No adverse events were reported. Gross-eyed evaluation was feasible in all cases and guided effective sampling. **Conclusions**: EUS-FNB with two puncture numbers and the fanning technique achieves high diagnostic performance for pancreatic malignancy without the need for ROSE and MOSE. Two passes appear sufficient in most cases, supporting a simplified and safe approach that minimizes unnecessary needle passes.

## 1. Introduction

Pancreatic ductal adenocarcinoma (PDAC) is a leading cause of cancer-related mortality worldwide, with a 5-year survival rate of only 11% in the United States [1]. The lethality of this disease underscores the importance of early and accurate diagnosis. Endoscopic ultrasound-guided fine-needle biopsy (EUS-FNB) has become the standard method for obtaining tissue from solid pancreatic masses (SPMs), offering high diagnostic accuracy with minimal invasiveness. Traditionally, EUS-guided tissue acquisition (EUS-TA) relied on fine-needle aspiration (FNA) in conjunction with rapid on-site cytopathologic evaluation (ROSE), which allows the real-time assessment of specimen adequacy and guides the number of needle passes. However, the routine use of ROSE has declined in many institutions due to its resource-intensive nature and limited availability of cytopathologists [2]. In response, the emergence of core biopsy techniques using novel needle designs—such as Franseen-tip or fork-tip needles—has significantly improved the tissue yield [3,4] and histologic quality, potentially obviating the need for ROSE [5,6,7]. Recent systematic reviews and meta-analyses have reported that these contemporary FNB needles can achieve a diagnostic accuracy comparable to or exceeding that of FNA with ROSE [8,9], particularly when a minimum of two to three passes are performed. Chalhoub et al. found that, while three passes improved the diagnostic parameters over one or two, additional passes beyond the third conferred minimal benefits [10]. Notably, their data suggested that two passes using modern Franseen- or fork-tip needles were sufficient to meet the American Society for Gastrointestinal Endoscopy (ASGE) and American College of Gastroenterology (ACG) benchmarks for diagnostic accuracy (≥70%) and tissue adequacy (≥85%) [11]. Accordingly, the latest European Society of Gastrointestinal Endoscopy (ESGE) technical and technology review recommends the preferential use of end-cutting FNB needles to sample solid pancreatic masses, especially in settings where rapid on-site evaluation (ROSE) is not available [12].

In parallel, macroscopic on-site evaluation (MOSE), where the visible white core length is used as a surrogate for histologic adequacy, and the gross-eyed method (white core tissue seen) have been proposed as practical alternatives to ROSE, particularly in resource-limited settings [13,14]. Touch imprint cytology (TIC) has also demonstrated comparable diagnostic performance to histology, further supporting the notion that non-ROSE methods can maintain diagnostic integrity [15].

Despite these developments, the optimal number of needle passes for EUS-FNB in the absence of ROSE, particularly when relying on the gross-eyed method, remains poorly defined in real-world clinical settings. Moreover, there are few studies evaluating this approach in dedicated cohorts of pancreatic malignancy.

This study aimed to assess the diagnostic rate of malignancy, yield rate of malignancy, and tissue adequacy of EUS-FNB in patients with pancreatic malignancy using a gross-eyed evaluation of tissue quality with the fanning technique and fewer needle passes (two instead of three), as fewer needle passes could be safer, with fewer adverse events. Determining the optimal number to balance diagnosis and side effects is important. Given emerging concerns regarding needle tract seeding after EUS-guided tissue acquisition [16,17,18], particularly in patients undergoing surgical resection, minimizing unnecessary passes has become increasingly important.

## 2. Materials and Methods

### 2.1. Study Design and Patient Selection

This retrospective single-center study was conducted from January 2022 to December 2025 at Tri-Service General Hospital in Taiwan, a tertiary referral hospital. The study was approved by the Institutional Review Board (IRB approval number: A202405188). Informed consent was waived given the retrospective nature of the research. Patients were included if they were ≥18 years of age and had a definite diagnosis of pancreatic malignancy. The exclusion criteria were (1) diagnosis made using non-EUS modalities, including surgical pathology or CT-guided biopsy; (2) diagnosis made according to lesions of metastatic sites other than the pancreas; or (3) EUS-FNB procedures with fewer than three needle passes.

### 2.2. EUS-FNB Procedure and MOSE Protocol

**Figure 1:**
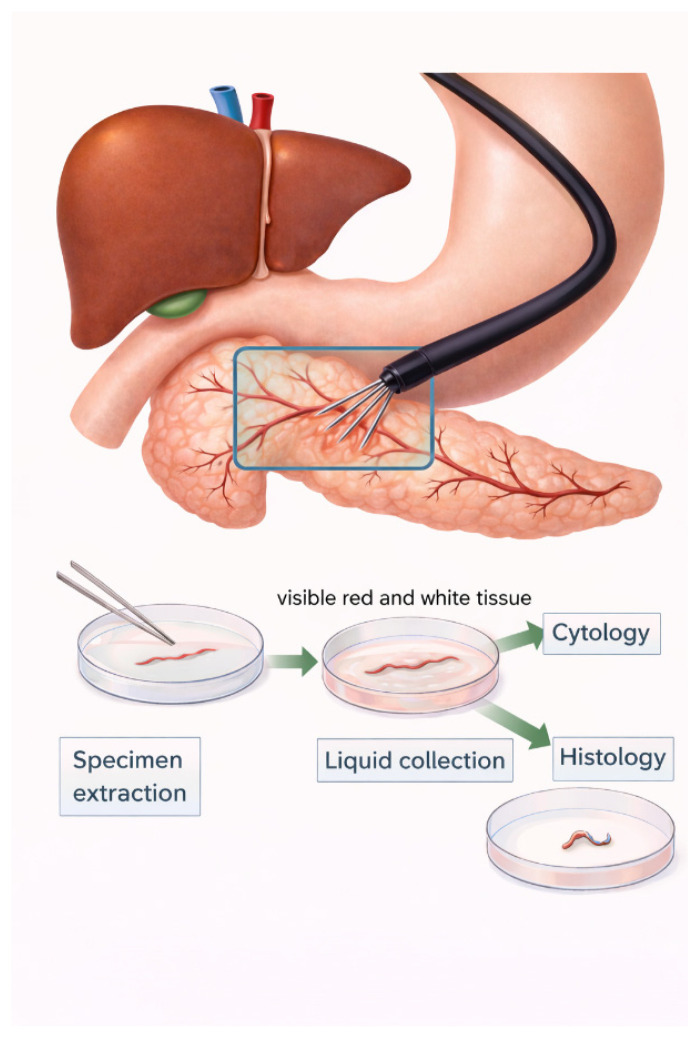
EUS-FNB fanning technique with gross visual assessment.

**Figure 2:**
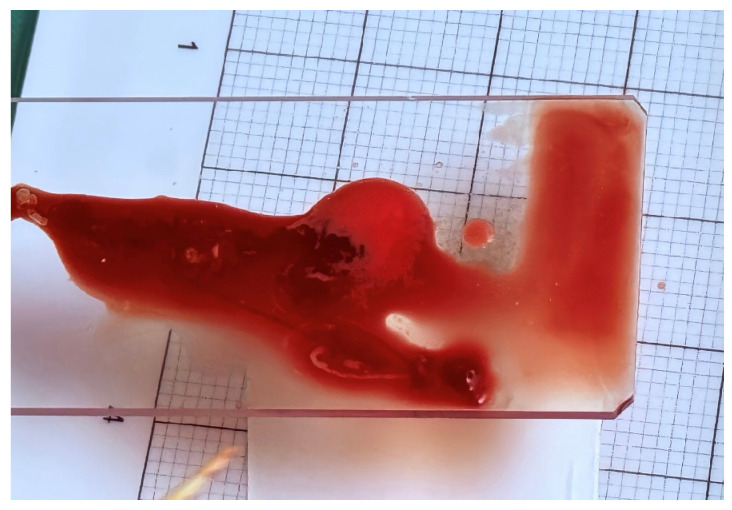
Specimen of red and white tissues.

All EUS-FNB procedures were performed using a linear-array echoendoscope (GF-UCT260; Olympus Medical Systems, Tokyo, Japan), with either a 22-gauge or 19-gauge needle. In accordance with the ESGE technical recommendations [12], the fanning technique was routinely applied during each needle pass to sample multiple areas of the lesion and maximize the diagnostic yield [19], and the procedures were conducted under conscious sedation. The needle choice and suction technique (slow-pull or standard syringe suction) were determined at the discretion of the endosonographer. Rapid on-site evaluation (ROSE) was not available during the study period. Instead, the specimen adequacy was assessed intra-procedurally using the gross-eyed method. After each pass, the aspirated specimen was expelled onto a piece of filter paper using the stylet. Gross-eyed evaluation was performed by the endoscopist, who visually inspected the sample for whitish worm-like tissue cores (Figure 1 and Figure 2). Regardless of whether the initial two passes yielded visible core or were composed predominantly of blood and clot, a third pass was routinely performed; no additional needle passes were made. All samples were immediately fixed in 10% neutral buffered formalin for histologic processing.

### 2.3. Final Diagnosis and Histologic Confirmation

The final diagnosis of malignancy was confirmed via histopathology. Pancreatic ductal adenocarcinoma, neuroendocrine tumors, GISTs, and pancreatic carcinomas were all classified as malignant. Cytologic diagnoses categorized as “malignant” or “suspicious for malignancy” were considered positive.

### 2.4. Outcome Measures

The primary outcome was the diagnostic rate of malignancy, defined as the proportion of cases in which the EUS-FNB diagnosis was concordant with the final histopathology. The secondary outcomes included tissue adequacy, defined as the presence of sufficient cellular or histologic material on histopathology to allow for a pathologic interpretation, regardless of whether a definitive diagnosis was achieved, and the yield rate of malignancy, defined as the proportion of cases in which EUS-FNB yielded a definitive diagnosis of malignancy or benignity on pathology. Cases with “atypical” or “non-diagnostic” reports were not counted as diagnostic.

### 2.5. Statistical Analysis

Continuous variables are expressed as the means ± standard deviation (SD). Categorical variables are presented as frequencies and percentages, and comparisons were performed using the Chi-square test. A *p*-value < 0.05 was considered statistically significant. Statistical analyses were performed using SPSS software version 24 (IBM Corp., Armonk, NY, USA).

## 3. Results

### 3.1. Patient Characteristics

**Figure 3:**
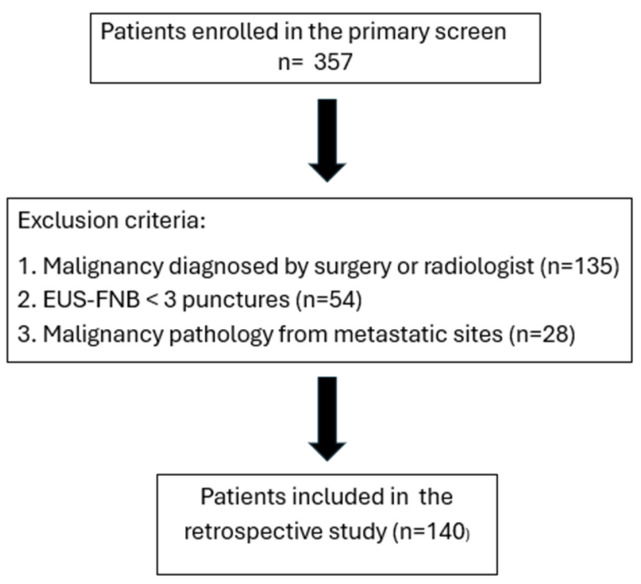
Flowchart of patient enrollment in this study.

A total of 357 patients were diagnosed with pancreatic malignancy during the study period. After applying the exclusion criteria—diagnosis established using non-EUS modalities (*n* = 135), EUS-FNB procedures with fewer than three needle passes (*n* = 54), or diagnosis based on a metastatic lesion (*n* = 28)—140 patients were included in the final analysis (Figure 3).

The mean age of the enrolled patients was 67.6 ± 11.2 years, and 49.3% were male. The majority of patients (81.4%) were diagnosed with pancreatic ductal adenocarcinoma (PDAC), followed by neuroendocrine tumors (10%), other neoplasms (6.4%), and carcinoma (2.1%). The PDAC group had a mean age of 69 ± 10.1 years, with 47.4% males. Detailed demographics and clinical features, including smoking, alcohol use, diabetes, viral hepatitis status, tumor location, disease stage, and tumor size, are summarized in Table 1.

Tumors were most commonly located in the pancreatic head (*n* = 68), followed by the body (*n* = 28), tail (22), uncinate process (*n* = 15), and neck (*n* = 7). The disease stage distribution was Stage I (*n* = 22), Stage II (*n* = 17), Stage III (*n* = 19), and Stage IV (*n* = 82). Lesions ≥20 mm accounted for 90.7% (*n* = 127) of cases.

### 3.2. Diagnostic Performance According to the Number of Passes

The diagnostic rate of malignancy of the EUS-FNB increased with the number of passes:

• One pass: 72.1%;
• Two passes: 82.9%;
• Three passes: 90.7%.

**Figure 4:**
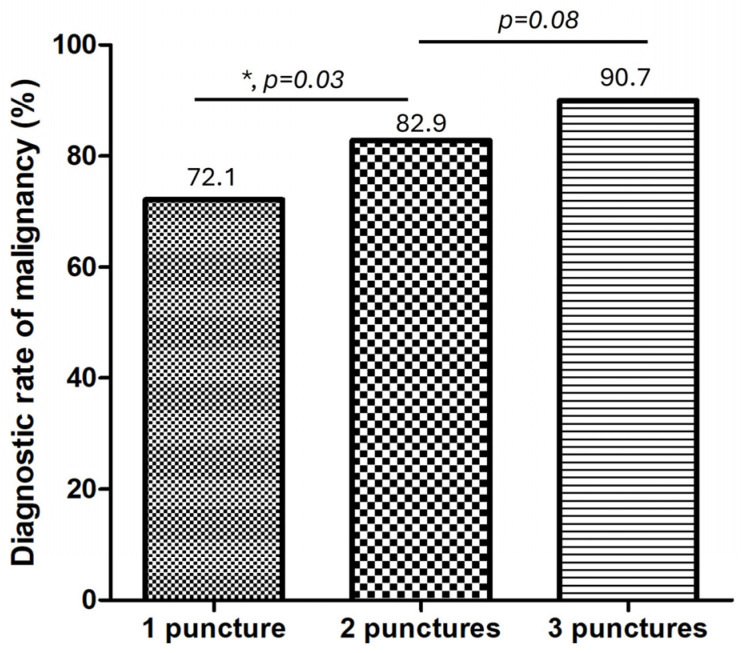
Diagnostic rate of malignancy according to the number of needle passes. * means significant with *p *< 0.05.

There was a significant improvement in the diagnostic rate of malignancy between one and two passes, but there was no meaningful gain between two and three passes (Figure 4). This pattern is consistent with prior prospective and pooled analyses, which demonstrated a plateau effect in diagnostic accuracy after the second pass when using modern FNB needles [20].

### 3.3. Tissue Adequacy

Tissue adequacy, defined as the histologic assessment of samples containing sufficient material for diagnosis, was high across all groups:

• One pass: 94.3%;
• Two passes: 99%;
• Three passes: 100%.

**Figure 5:**
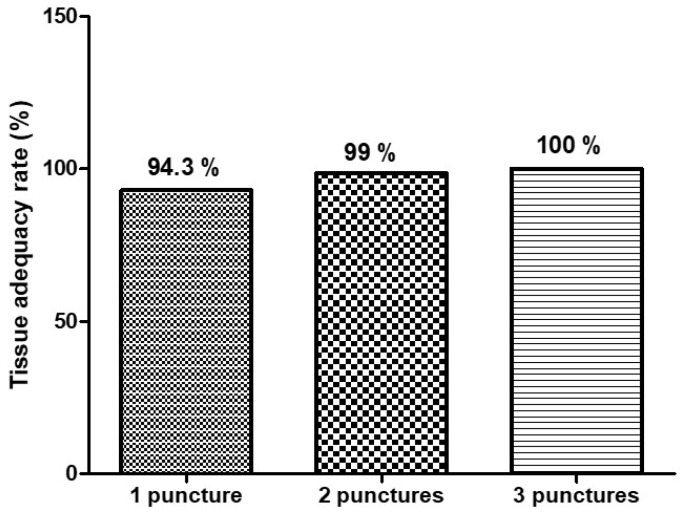
Tissue adequacy rate according to the number of needle passes.

Although the adequacy increased incrementally, all groups exceeded the benchmark of 85% proposed by ASGE for EUS-guided sampling quality. The majority of patients reached tissue adequacy within two passes based on the gross-eyed evaluation (Figure 5), supporting the role of the gross-eyed method in reducing unnecessary punctures.

### 3.4. Yield Rate of Malignancy

The overall diagnostic yield—defined as the proportion of cases yielding a definitive benign or malignant diagnosis—mirrored the diagnostic rate trend:

• One pass: 82.1%;
• Two passes: 89.2%;
• Three passes: 91.4%.

**Figure 6:**
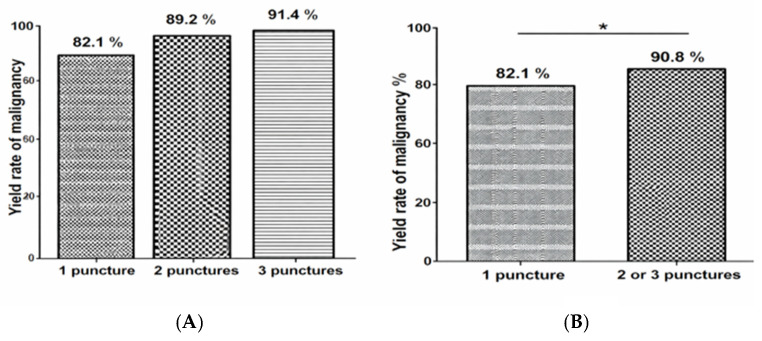
Yield rate of malignancy according to the number of needle passes. (**A**) Comparison of the yield rate of malignancy according to the number of needle passes. (**B**) Comparison of the yield rate of malignancy between one pass and two or three passes. * means significant with *p *< 0.05.

A small subset of cases remained “non-diagnostic” or “atypical” despite adequate tissue, highlighting the interpretative limitations of cytology alone. The yield rate of malignancy improved significantly from one pass to two or three passes (Figure 6), which aligns with prior randomized trials comparing EUS-FNB with and without ROSE [21].

### 3.5. Annual Trends in the Diagnostic Rate of Malignancy of EUS-FNB According to the Number of Needle Passes

**Figure 7:**
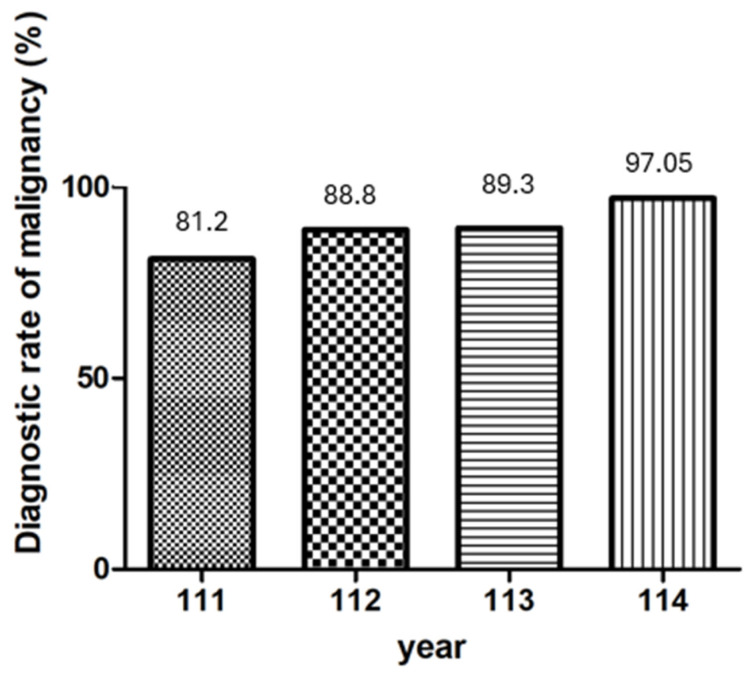
Annual trends in the diagnostic rate of malignancy of EUS-FNB according to the number of needle passes.

The diagnostic rate of malignancy of EUS-FNB demonstrated a progressive increase across three years, irrespective of the number of needle passes, and the diagnostic rate improved from 81.2% to 97.05% overall (Figure 7). This upward trend suggests enhanced procedural performance over time.

### 3.6. Univariable and Multivariable Analysis of Misdiagnosis at One Pass

No significant association was found between misdiagnosis at one pass and the tumor location (head vs. body/tail, *p* = 0.24), tumor size (≥20 mm vs. <20 mm, *p* = 0.77), or needle type (19G vs. 22G, *p* = 0.32). The details of factors associated with misdiagnosis at one puncture are summarized in Table 2.

### 3.7. Safety and Procedural Metrics

No adverse events, such as pancreatitis, bleeding, or infection, were reported in the 106 analyzed cases. All procedures were completed without interruption, and the gross-eyed method was feasible in 100% of cases.

## 4. Discussion

EUS-FNB with the fanning technique is superior to the standard approach to obtain tissue for diagnosis [22]. In our previous experience, even without the use of MOSE, the diagnostic rate was still high. To address this issue, all procedures were performed using the fanning technique, and we evaluated the sample quality using gross visual (gross-eyed) assessment in the absence of ROSE and MOSE.

The diagnostic rate of malignancy improved from 72.1% with one pass to 82.9% with two passes and reached 90.7% with three passes. This plateau beyond the second pass is consistent with the prior literature [10]. Chalhoub et al. reported a pooled diagnostic accuracy of 91.5% for pancreatic lesions with three or more passes but also found that modern FNB needles often achieved benchmark tissue adequacy (≥85%) with just two passes [10]. Our findings similarly suggest that increasing the number of passes beyond two may not meaningfully improve the diagnostic outcomes when a gross-eyed method is employed to guide tissue acquisition, which is highly consistent with the latest ESGE technical review recommending two passes when using end-cutting FNB needles in the absence of ROSE [12]. This concordance supports the concept that the modern FNB needle design, combined with optimized sampling techniques, allows procedural simplification without compromising diagnostic performance.

In line with this, a longitudinal analysis of our institutional data revealed a consistent year-over-year improvement in the diagnostic rate of malignancy across all passes. These trends suggest that, beyond procedural standardization, accumulated operator experience, refined tissue handling techniques, and possibly improvements in the needle design or gross-eyed evaluation all contribute to better diagnostic outcomes over time.

Importantly, the tissue adequacy in our cohort was high across all groups (94.3% for one, 99% for two, and 100% for three passes), exceeding ASGE thresholds and aligning with recent randomized controlled trials evaluating MOSE [23]. Mangiavillano et al. demonstrated in a large multicenter RCT that EUS-FNB with MOSE had non-inferior diagnostic accuracy compared to conventional three-pass FNB [24], while significantly reducing the number of required punctures. In addition, the ESGE emphasizes that ROSE is not routinely required for EUS-FNB of solid pancreatic lesions, particularly when end-cutting needles and macroscopic assessment strategies are employed [12]. Our results extend this recommendation by demonstrating that a gross-eyed evaluation-guided two-pass protocol can maintain high diagnostic accuracy and tissue adequacy even in centers without cytopathology support.

The gross-eyed technique we employed relies on the real-time visual inspection of visible white core fragments. This method is simple, cost-effective, and well-suited to centers lacking cytopathology support. While the presence of visible core does not always guarantee diagnostic yield, our findings indicate that its use as a surrogate for tissue adequacy was highly reliable. Nevertheless, gross-eyed evaluation remains a subjective and operator-dependent method. Although the specimen adequacy was confirmed post hoc via the histopathology in our study, future studies are required to standardize gross-eyed criteria and to validate this approach against objective measurements, such as the visible core length or quantitative histologic core content, ideally in prospective multicenter settings.

From a practical standpoint, the proposed two-pass gross-eyed-guided EUS-FNB protocol may be particularly suitable for centers with limited access to ROSE, cytopathology support, or advanced imaging modalities. By relying on modern end-cutting FNB needles, standardized sampling techniques such as the fanning technique, and simple visual assessment, this approach may facilitate broader implementation across institutions with varying levels of equipment and resources.

The selection of the suction technique during EUS-guided tissue acquisition in the present study was left to the discretion of the endosonographer, reflecting real-world clinical practice. This approach is supported by a recent network meta-analysis by Giri et al. [25], which compared dry suction, wet suction, slow-pull, and no suction techniques; they found no single method to be consistently superior in terms of the diagnostic accuracy or tissue adequacy when modern EUS-guided sampling techniques were applied. These findings suggest that the suction strategy can be individualized based on the lesion characteristics, such as vascularity or bleeding tendency, rather than dictated by a universally optimal method.

Furthermore, our yield rate of malignancy (proportion of cases with definitive malignant or benign diagnosis) closely mirrored the diagnostic rate of malignancy, reaffirming the utility of two passes in high-performing centers with experienced operators. The minimal rate of inadequate samples or indeterminate diagnoses also supports the robustness of the gross-eyed assessment in determining when to cease sampling.

The safety profile of our cohort was favorable, with no reported adverse events. This is particularly relevant in the context of pass minimization. Excessive punctures have been associated with increased complication risks, including bleeding and post-procedural pancreatitis. Our strategy minimizes unnecessary needle passes and aligns with principles of patient safety and procedural efficiency.

From a broader procedural perspective, ancillary techniques such as contrast-enhanced harmonic endoscopic ultrasound (CH-EUS)-guided tissue acquisition have been proposed to further optimize lesion targeting and specimen quality in selected cases [26]. Prior studies have demonstrated that CH-EUS-guided fine-needle aspiration (CH-EUS-FNA) improves the diagnostic accuracy compared with conventional B-mode EUS-FNA by facilitating sampling from viable tumor regions while avoiding necrotic or fibrotic areas [27,28]. However, emerging evidence suggests that although CH-EUS-FNA outperforms standard FNA, its diagnostic performance may still be inferior to that achieved with contemporary end-cutting FNB needles, such as Franseen- or fork-tip designs, for which the reported diagnostic accuracy can approach 90–95% in pancreatic solid lesions [29]. In this context, advances in needle design may play a more decisive role in tissue acquisition than imaging enhancement alone. Although CH-EUS was not routinely used in the present study, these findings highlight a complementary role for advanced imaging techniques alongside modern FNB needles and gross-eyed assessment, particularly in challenging lesions or heterogeneous tumors.

From a biological standpoint, our use of gross-eyed assessment gains additional validation from a recent study by Lin et al., which investigated the biological content of red material obtained during EUS-FNB [30]. While traditional MOSE emphasizes measurement of white core length to assess specimen adequacy [31,32], Lin’s study demonstrated that red material—often abundant but traditionally disregarded—contains a significantly higher amount of tumor DNA and shows high concordance in K-ras mutation profiles compared to white cores. These findings suggest that even when white cores are small or not clearly visible, red material should not be dismissed as merely blood contamination but rather recognized as a potentially rich source of tumor content, particularly for molecular analysis. Such results support the validity of the gross-eyed method, which does not require millimeter-scale measurements of white core length but rather depends on the visual identification of tissue. Compared with the findings reported by Lin et al., the high yield rate of malignancy achieved in our cohort suggests that gross-eyed evaluation may capture biologically meaningful tissue, including red material with potential molecular relevance, even when classical MOSE criteria are not strictly met.

Furthermore, minimizing the number of passes aligns with safety considerations. Needle tract seeding, while rare, is an increasingly recognized risk of EUS-guided procedures. Studies have reported cases of tumor seeding along the needle tract, particularly in patients undergoing distal pancreatectomy or subsequent curative surgery [16,17,18]. By establishing a two-pass protocol with reliable gross-eyed assessment, clinicians can balance the diagnostic rate of malignancy and tissue adequacy while mitigating the procedural risks.

This study has several limitations. First, it was a retrospective single-center analysis with a relatively limited sample size, which may introduce selection bias, limit the statistical power, and restrict the generalizability of the findings to broader patient populations or different practice settings. Second, the gross-eyed assessment was not validated against objective core length measurements or histologic core content, although the adequacy was confirmed post hoc via pathology. Third, the inter-operator variability in assessing macroscopic adequacy was not formally analyzed. Lastly, the inclusion of only patients who underwent at least three needle passes may have introduced a selection bias. This design could potentially overestimate the diagnostic performance of one- or two-pass strategies, as cases with early diagnostic failure after fewer passes were not represented. Therefore, caution is warranted when extrapolating our findings to true minimal-pass EUS-FNB protocols. Future prospective multicenter randomized controlled studies are warranted to validate these findings and to further define the optimal sampling strategy across diverse clinical settings.

## 5. Conclusions

In patients undergoing EUS-FNB with the fanning technique for pancreatic malignancy without ROSE, two needle passes guided by gross-eyed evaluation provide optimal diagnostic performance. Additional passes beyond two confer minimal benefits and may be unnecessary in most clinical settings. The gross-eyed method is a practical and effective alternative to ROSE and supports a simplified, cost-effective, and safe strategy for tissue acquisition in pancreatic malignancy diagnosis.

## Figures and Tables

**Table 1:** Patient characteristics.

Characteristics	PDAC	Carcinoma	Neuroendocrine Tumor	Others
Patient number (%)	114 (81.4)	3 (2.1)	14 (10)	9 (6.4)
Patient age (yrs)	69.0 ± 10.1	66.7 ± 14	55.4 ± 11.7	64.7 ± 14.6
Gender (male)	54	2	8	5
Smoking, no. (%)	22 (19.3)	1 (33.3)	2 (14.3)	0 (0)
Alcohol, no. (%)	17 (14.9)	1 (33.3)	0 (0)	0 (0)
Diabetes mellitus, no. (%)	54 (48.9)	2 (66.7)	2 (14.3)	4 (44.4)
Family history, no. (%)	3 (2.6)	0 (0)	0 (0)	1 (11.1)
HBV, no. (%)	40 (35.1)	1 (33.3)	6 (42.9)	0 (0)
HCV, no. (%)	5 (4.4)	0 (0)	0 (0)	0 (0)
Tumor location				
Head	57	0	5	6
Neck	5	0	0	2
Uncinate process	11	0	3	1
Body	22	1	5	0
Tail	19	2	1	0
Disease stage				
Stage I	17	0	3	2
Stage II	12	1	4	0
Stage III	18	0	0	1
Stage IV	67	2	7	6
Tumor size				
≥20 mm	107	3	10	7
<20 mm	7	0	4	2

**Table 2:** Factors associated with misdiagnosis at one puncture.

			Univariable Analysis	Multivariate Analysis
			*p*-Value	*p*-Value
Location	Ph	68	0.65	0.34
	Pbt	72		
Size	≥20 mm	127	0.35	0.77
	<20 mm	13		
Needle	19G	10	0.12	0.32
	22G	130		

## Data Availability

The data presented in this study are available on request from the corresponding authors due to restrictions of patient privacy.

## Abbreviations

The following abbreviations are used in this manuscript:

EUS-FNB	Endoscopic ultrasound-guided fine-needle biopsy
SPMs	Solid pancreatic masses
ROSE	Rapid on-site cytopathologic evaluation
PDAC	Pancreatic ductal adenocarcinoma
MOSE	Macroscopic on-site evaluation
TIC	Touch imprint cytology
